# Retraction Note: Circular RNA circRNF20 promotes breast cancer tumorigenesis and Warburg effect through miR-487a/HIF-1α/HK2

**DOI:** 10.1038/s41419-026-08545-z

**Published:** 2026-03-20

**Authors:** Lili Cao, Min Wang, Yujin Dong, Bo Xu, Ju Chen, Yu Ding, Shusheng Qiu, Liang Li, Elena Karamfilova Zaharieva, Xinwen Zhou, Yanbin Xu

**Affiliations:** 1 Department of Oncology, Zibo Central Hospital, Zibo, 255020 China; 2 Department of Ultrasound, Zibo Central Hospital, Zibo, 255020 China; 3 Department of Breast and Thyroid Surgery, Zibo Key laboratory of Breast cancer Individualized diagnosis, treatment and transformation, Zibo Central Hospital, Zibo, 255020 China; 4 Department of Experimental Oncology, Research Institute for Radiation Biology and Medicine, Hiroshima University, Hiroshima, 734-8553 Japan; 5 School of Radiation Medicine and Protection, Medical College of Soochow University, Suzhou, 215123 China

Retraction Note to: *Cell Death & Disease* 10.1038/s41419-020-2336-0, published online 24 February 2020

The Editors-in-Chief have retracted this article.

After publication, concerns were raised on images presented in this article for overlaps with itself and another retracted article [1].

Specifically:

In Fig. 2C: Panel sh-NC and circRNF20OE appear to overlap.

In Fig. 2C: Panel Vector and sh-circRNF20 appear to overlap.

Also, Image sh-circRNF20 from Fig. 2C of this article and Image NC from Fig. 2G of the retracted article [1] appear to be similar.

This and the retracted article were submitted within a close timeframe, however there are no common authors. The authors were contacted to clarify the issues but they were unable to provide the complete raw data. The Editors therefore have lost confidence in the data presented in this article.

The authors have not responded to correspondence from the Publisher about this retraction.
